# Involvement of the Actin Machinery in Programmed Cell Death

**DOI:** 10.3389/fcell.2020.634849

**Published:** 2021-02-09

**Authors:** Weida Ren, Wanyu Zhao, Lingbo Cao, Junqi Huang

**Affiliations:** Key Laboratory for Regenerative Medicine, Ministry of Education, College of Life Science and Technology, Jinan University, Guangzhou, China

**Keywords:** programmed cell death, actin, actin-binding proteins, actin-modulating proteins, actin machinery, cytoskeleton

## Abstract

Programmed cell death (PCD) depicts a genetically encoded and an orderly mode of cellular mortality. When triggered by internal or external stimuli, cells initiate PCDs through evolutionary conserved regulatory mechanisms. Actin, as a multifunctional cytoskeleton protein that forms microfilament, its integrity and dynamics are essential for a variety of cellular processes (e.g., morphogenesis, membrane blebbing and intracellular transport). Decades of work have broadened our knowledge about different types of PCDs and their distinguished signaling pathways. However, an ever-increasing pool of evidences indicate that the delicate relationship between PCDs and the actin cytoskeleton is beginning to be elucidated. The purpose of this article is to review the current understanding of the relationships between different PCDs and the actin machinery (actin, actin-binding proteins and proteins involved in different actin signaling pathways), in the hope that this attempt can shed light on ensuing studies and the development of new therapeutic strategies.

## Introduction

Cell death, according to the recommendations of the Nomenclature Committee on Cell Death, can be classified into two big categories: accidental cell death (ACD) and programmed cell death (PCD) (Galluzzi et al., 2018). ACD is triggered by unexpected injury without controllable molecular machinery. PCD, on the contrary, defines genetically fine-regulated preset cell death processes. PCD is being intensively studied conferred by its anti-cancer pharmacological potential and physiological functions during development. To date, a handful of conceptually distinct PCDs have been discovered (Tang et al., 2019). According to the timeline of naming, PCDs include: Apoptosis, Lysosomal cell death, Pyroptosis, NETosis, Necroptosis, Entosis, Parthanatos, Ferroptosis, Autosis, Alkaliptosis, Oxeiptosis, etc. These subprograms can exterminate cells in different ways, causing featured morphological alterations, signaling cascade changes and different immunological consequences.

Actin, being one of the most abundant proteins in cells, is evolutionarily conserved across kingdoms. Similar to other cytoskeleton proteins, actin exists as free monomer called G-actin (spherical) or as part of linear double helical polymeric microfilaments called F-actin (filamentous) (Holmes et al., 1990). Both G-actin and F-actin are found in the cytoplasm and the nucleus. The cellular actin cytoskeleton is accounted for virtually most activities of cellular functions, such as gene transcription, protein translation, cell morphogenesis, membrane dynamics and cell mechanics. To cope with different physiological or pathological stimulations, the spatial and temporal dynamics of the actin cytoskeleton change rapidly. Disruption of the localization, balance or dynamics of the actin pool correlates with diverse diseases, ranging from aging to cancer. Actin, actin-binding proteins and actin-modulating proteins constitute a broader concept which we define as “actin machinery” in this review. A significant fraction of actin function lies within the activities of a category of proteins called actin-binding proteins. Actin-binding proteins refer to an array of proteins which directly binds to G-actin or F-actin, include, but are not limited to Gelsolin, Cofilin, the Arp2/3 complex, etc. Actin-modulating proteins depict proteins interfering actin cytoskeleton functions in different actin signaling pathways, albeit do not directly bind to actin (RhoA, ROCK, Cdc42, etc.). The faithful execution of actin cytoskeleton function is a consequence of the interplays between proteins within the actin machinery.

Lines of evidences, some of which are compelling, have indicated that PCDs may be closely correlated with the actin machinery (Gourlay and Ayscough, 2005; Franklin-Tong and Gourlay, 2008; Smertenko and Franklin-Tong, 2011). However, how exactly actin machinery functions in different PCDs and whether these mechanisms exhibit similarities are still enigmatic. Currently, the relationship between PCDs and the actin machinery has not been extensively reviewed.

Here, in this review, we attempt to address the relationship between PCDs and the actin machinery by summarizing existing literatures. For clarity, the following content will be separated into 11 parts, according to the time of discovery of each PCD.

## Apoptosis

**Figure 1:**
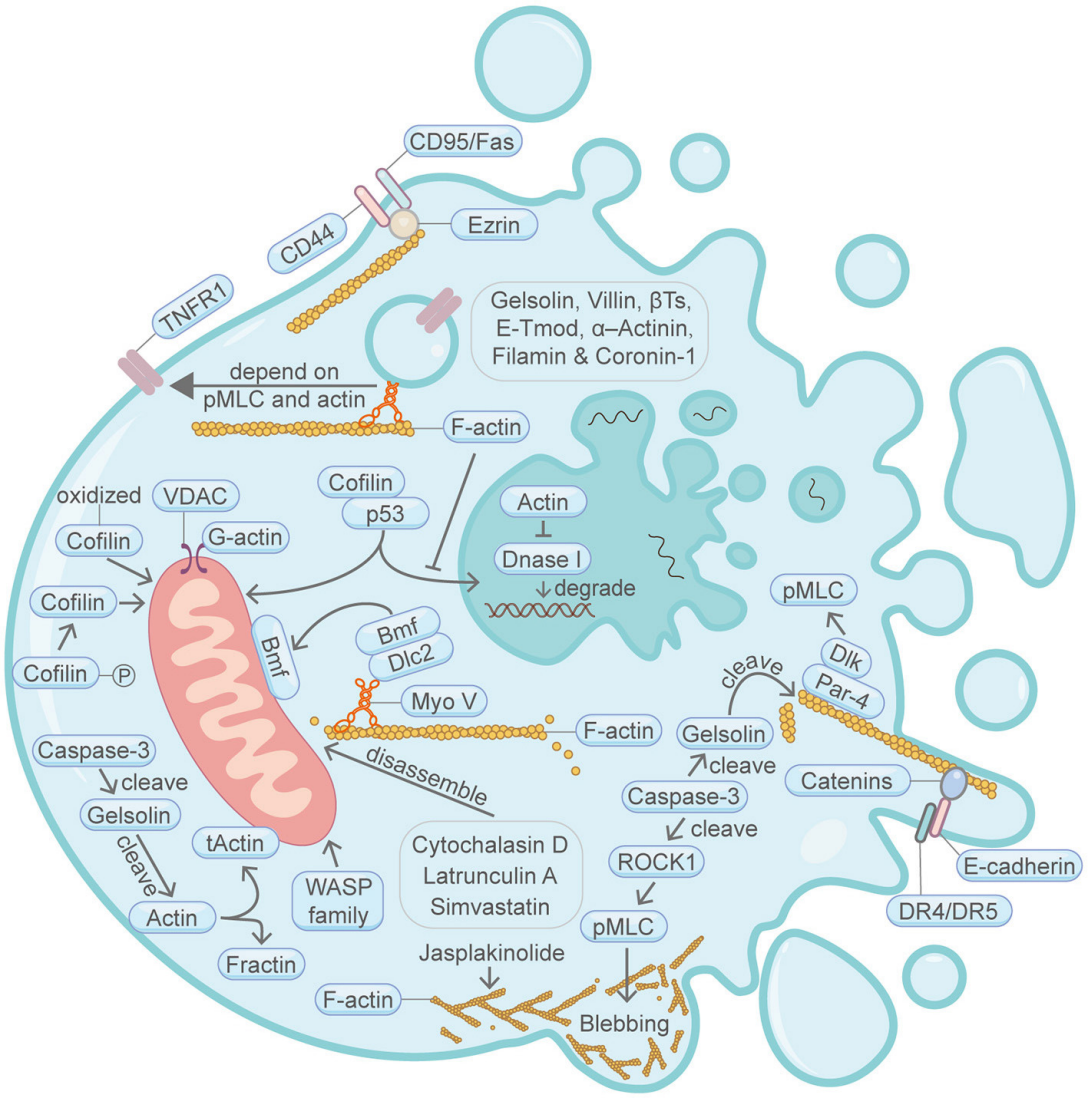
Schematic illustrating apoptosis and the actin machinery. Actin-binding proteins, such as Gelsolin, Villin, β-Thymosins, E-tropomodulin, Filamin and Coronin-1, play active roles in apoptosis. The actin cytoskeleton integrity is essential for CD95/Fas-mediated apoptosis. During another TNF-induced apoptosis, plasma membrane translocation of the TNFR1 requires myosin II motor and actin. E-cadherin and catenins engagement can augment apoptosis activation by linking DR4/DR5 to the F-actin cytoskeleton. Bmf translocation from the filamentous actin to the mitochondria is important for apoptosis. Cofilin protein amount and its posttranslational modification status are important for apoptosis. Cytochalasin D, latrunculin A and Simvastatin can induce apoptosis through disrupting the actin cytoskeleton network. Cofilin and actin affects p53-mediated control of apoptosis. Par-4 can recruit Dlk to the filamentous actin, thereby enhancing the phosphorylation of MLC and induction of apoptosis. WASP family protein WAVE1 can regulate apoptosis through affecting mitochondria. Actin is cleaved by caspase during apoptosis, resulting in the production of tActin and Fractin. tActin, rather than Fractin, can specifically induce morphological changes resembling apoptosis. Actin is also involved in the regulation of DNA degradation during apoptosis. Membrane blebbing is supervised by actomyosin contractility, which in turn is regulated by Caspase-3-ROCK1 cleavage-pMLC axis. Please see the main text for more detailed information. Abbreviations: Caspase, cysteine aspartic protease; βTs, β-Thymosins; E-Tmod, E-tropomodulin; TNFR1, TNF receptor-1; VDAC, voltage-dependent anion channel; Bmf, Bcl2-modifying factor; Dlc2, dynein light chain 2; pMLC, phosphorylated myosin light chain; Dlk, DAP like kinase; Par-4, prostate apoptosis response-4; DR4/DR5, death receptor 4/5; ROCK1, Rho-associated coiled-coil kinase; Myo V, myosin V; WASP, Wiskott-Aldrich Syndrome protein; tActin, mitochondria-targeted N-myristoylated 15 kDa fragment of actin; Fractin, N-terminal 32 kDa fragment of actin; Dnase I, deoxyribonuclease I.

It is widely appreciated that apoptosis is the most well-studied cell death programme. Among the 11 PCDs we will focus on in this review, apoptosis was discovered the earliest. In the process of apoptosis, various independent signaling pathways triggered by extrinsic or intrinsic stimuli lead to the activation of cysteine-aspartic proteases (caspases) (Strasser et al., 2000). Caspases cleave many intracellular substrates, thereby gradually causing cell shrinkage, chromatin condensation, nuclear fragmentation, plasma membrane blebbing/blistering, apoptotic bodies formation and other apoptosis-specific features (Earnshaw et al., 1999; Nicholson, 1999; Fuentes-Prior and Salvesen, 2004). A myriad of evidences has tightly connected apoptosis and the actin machinery (Figure 1) (Gourlay and Ayscough, 2005; Moss and Lane, 2006; Desouza et al., 2012). For example, at the tissue level, in epithelial monolayers, dying cells destined for apoptosis are extruded from the monolayer by assembly of contractile F-actin and myosin structures in neighboring healthy cells (Rosenblatt et al., 2001). At the cellular level, the actin cytoskeleton is reorganized into a peripheral actomyosin cortical ring during apoptosis (Ndozangue-Touriguine et al., 2008). Moreover, disruption of the actin-binding protein α-Actinin at the focal adhesion renders cells vulnerable to apoptosis (Triplett and Pavalko, 2006). Similarly, actin-binding proteins, such as Gelsolin, β-Thymosins, E-tropomodulin, Filamin and Coronin-1, are found to play essential roles in apoptosis (reviewed in detail elsewhere) (Franklin-Tong and Gourlay, 2008; Desouza et al., 2012). Intriguingly, certain actin machinery proteins are found to be important substrates for caspases and actin plays vital roles in the initiation and execution steps of apoptosis (Moss and Lane, 2006). To further illustrate the connection between apoptosis and the actin machinery, we will elaborate on different stages of apoptosis such as the induction period and the execution period in the subsequent paragraphs.

### Induction Period

During apoptosis induction period, various death commands are introduced into cells, mainly through the extrinsic pathway and the intrinsic pathway. Actin is an important regulator or target in both pathways.

In the extrinsic apoptotic pathway, it has been demonstrated that the aggregation of CD95/Fas (belongs to the tumor necrosis factor (TNF) receptor superfamily) and CD44 death receptors requires actin, which is instrumental for stimulating these death receptors to elicit the downstream apoptotic responses in Jurkat cells (Franklin-Tong and Gourlay, 2008). It is found that an actin-binding protein Ezrin interfaces between CD95/Fas and F-actin, thereby activating apoptosis signaling (Parlato et al., 2000). Consistently, down regulation of ezrin and overexpressing Heat Shock Protein 70 together promote apoptosis (Yao et al., 2015). Moreover, during another TNF-induced apoptosis, plasma membrane translocation of the TNF receptor-1 is regulated by the actin-binding protein myosin II motor activity (Jin et al., 2001). Another study showed that E-cadherin and catenins engagement can augment apoptosis activation by linking DR4/DR5 to the F-actin cytoskeleton (Lu et al., 2014).

In the intrinsic apoptotic pathway, under physiological conditions, the pro-apoptotic factor bcl-2 family protein Bmf associates with the cytoplasmic F-actin networks through dynein light chain 2 (Dlc2) and myosin V protein complex (Puthalakath et al., 2001). When the adhesion to the F-actin cytoskeleton is disturbed under certain cellular stress such as UV radiation, Bmf will dissociate and translocate to the mitochondria, which then instigates pore formation in the outer mitochondrial membrane, ultimately initiating apoptosis (Grespi et al., 2010). In line with the function of Bmf translocation during apoptosis, Simvastatin, a cholesterol-lowering medication, can induce apoptosis through the disruption of F-actin integrity via the impairment of the actin regulatory protein small GTPase RhoA and Rac-1 (Kang et al., 2016). In another study, WAVE1, another WASP-family protein, can also regulate apoptosis through modulating Bcl-2 family protein Bcl-xL (Cheng et al., 2007). After induction of apoptosis by conditions such as oxidative stress or expressing a dephosphorylated Cofilin (active conformation that severs F-actin), the oxidized or dephosphorylated Cofilin translocates to the mitochondria before the subsequent release of cytochrome c (Chua et al., 2003; Klamt et al., 2009). Oppositely, down regulation of Cofilin abrogates cytochrome c release and apoptosis. Importantly, the apoptosis-inducing ability of Cofilin relies on its functional actin-binding domain. Moreover, the application of the actin depolymerization agents cytochalasin D or latrunculin A can induce mitochondrial-dependent apoptosis, probably also through releasing Bmf from the filamentous actin and the myosin V motor (Martin and Leder, 2001; Puthalakath et al., 2001; Paul et al., 2002). During apoptosis, actin is also directly involved in the regulation of voltage-dependent anion channels (VDACs) (Gourlay and Ayscough, 2005). The opening and closing of VDACs change the permeability of the outer mitochondrial membrane and regulate the release of pro-apoptotic factors. In line with this, the F-actin stabilization agent jasplakinolide can elicit apoptosis in various cell lines (Odaka et al., 2000; Aida et al., 2016). Confusingly, another actin stabilization agent phalloidin which shares the same actin-binding site as jasplakinolide, is found to reduce cisplatin-mediated apoptotic cell death in primary cultures of porcine proximal tubular kidney cells (Kruidering et al., 1998).

When DNA is damaged, p53 (TP53) enters and accumulates within the nucleus, thereby eliciting apoptosis. Interestingly, p53 nuclear translocation can be attenuated by the increase of actin polymerization in the cytosol, thereby mitigating p53-triggered apoptosis (Wang et al., 2013). Consistently, it is found that activated Cofilin coopts p53 and promotes p53 mitochondrial and nuclear localization, resulting in promotion of apoptosis (Liu et al., 2017).

### Execution Period

Apoptosis execution period is the convergence point of extrinsic, intrinsic and other apoptosis-inducing pathways, culminate with a series of morphological and biochemical changes that define apoptosis, such as membrane blistering, chromatin condensation and DNA fragmentation, cell shrinkage, formation of apoptotic bodies (Mills et al., 1999).

Two homologous actin-binding proteins, Gelsolin and Villin, render cells insensitive to apoptosis by preserving actin dynamics (Wang et al., 2012). At this stage, Caspase-3 cleaves Gelsolin and the cleaved Gelsolin fragment then subsequently cleaves the filamentous actin (Kothakota et al., 1997), resulting in the production of an N-terminal 32 kDa fragment (Fractin) and a mitochondria-targeted N-myristoylated 15 kDa fragment (tActin) (Utsumi et al., 2003). Expression of tActin, rather than Fractin, can specifically induce morphological changes resembling apoptosis (Mashima et al., 1999). Strikingly, the above-mentioned tActin-mediated apoptosis kills cells without obviously activating caspases, supporting the idea that actin functions as a downstream mediator in apoptosis.

Actin is also involved in the regulation of DNA degradation during apoptosis. G-actin monomer interacts with deoxyribonuclease I (DNase I) with high affinity and inhibits its activity (Weber et al., 1994). Correspondingly, DNase I is responsible for the degradation of nuclear DNA strands upon apoptosis (Peitsch et al., 1993; Eulitz and Mannherz, 2007).

The actomyosin system is key to the structural and morphological changes during the execution period of apoptosis. Membrane blebbing, as an important morphological feature of apoptosis, is supervised by actomyosin contractility, which in turn is regulated by Caspase-3-ROCK1 cleavage-pMLC axis (Coleman et al., 2001). Myosin light chain (MLC) can also be phosphorylated through Par-4/Dlk (Vetterkind et al., 2005). Coexpression of prostate apoptosis response-4 (Par-4) and DAP like kinase (Dlk) initiates apoptosis. In the cytoplasm, Par-4 binds to the actin cytoskeleton, thereby interacting with Dlk, which can then phosphorylate its substrate, MLC, therefore leading to intense contraction of the actomyosin system and apoptosis (Vetterkind et al., 2005).

Collectively, the above overwhelming data highlight the essentiality of the actin machinery in apoptosis.

## Lysosomal Cell Death

**Figure 2:**
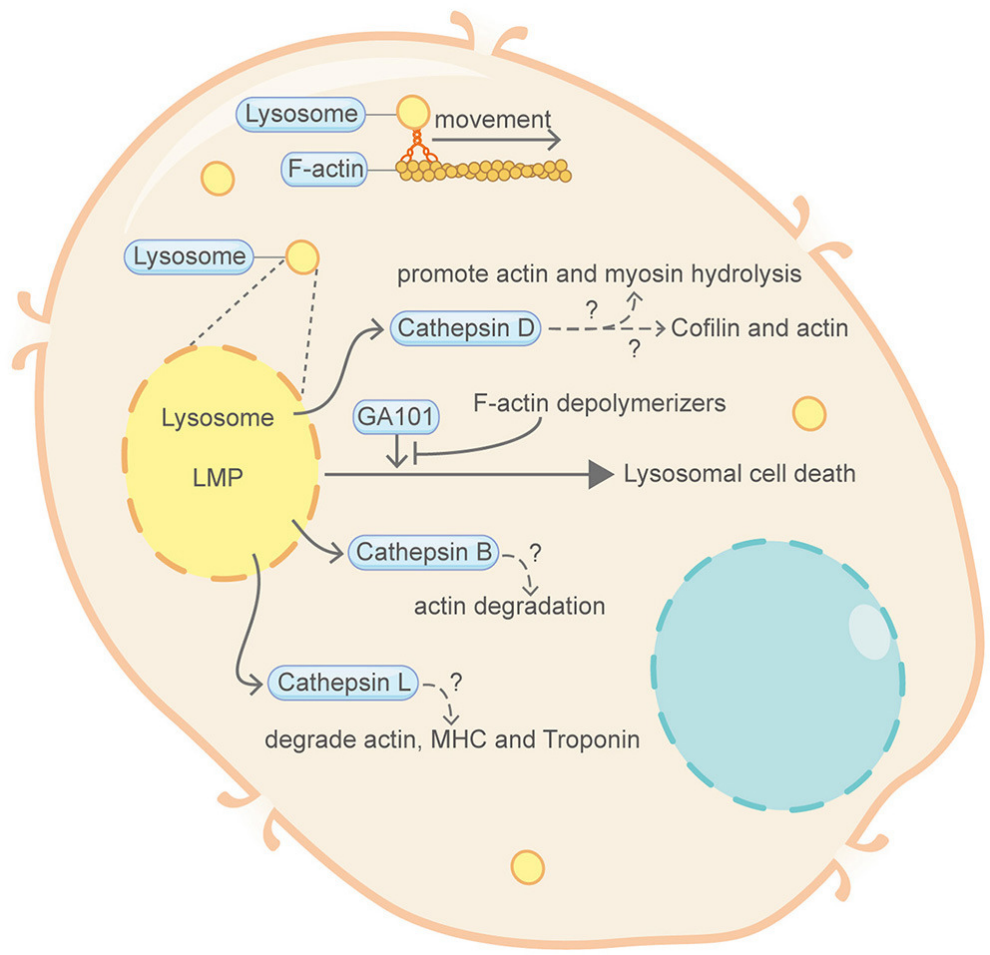
Schematic illustrating lysosomal cell death and the actin machinery. Permeabilization of lysosomal membrane and the subsequent release of hydrolytic enzymes including cathepsins are key features of lysosomal cell death. Direct evidence connecting lysosomal cell death and the actin machinery is limited. Nevertheless, certain proteins of the actin machinery, such as actin, myosin and Cofilin, can be degraded or modulated by cathepsins. Lysosome movement also closely correlates with F-actin and myosin. GA101, a type II CD20-targeted monoclonal antibody, can induce lysosomal cell death. This GA101-induced cell death can be abrogated by inhibitors of actin polymerization. Please see the main text for more detailed information. Abbreviations: LMP, lysosomal membrane permeabilization; MHC, myosin heavy chain.

Lysosomes are acidic organelles and intracellular recycling machines filled with numerous hydrolytic enzymes that can degrade a wide variety of structurally diverse materials, such as macromolecules, organelles and pathogens (Tang et al., 2019). Lysosomal membrane permeabilization (LMP) will result in the release of cathepsins (a large family of cysteine peptidases) and other hydrolytic enzymes from the collapsed lysosomal compartment into the cytoplasm, leading to lysosomal cell death (Figure 2). Lysosomal cell death can be induced by a number of stimuli, including but not limited to reactive oxygen species (ROS), lysosomotropic compounds and certain endogenous cell death effectors (Boya and Kroemer, 2008).

Direct involvement of the actin machinery in lysosomal cell death is limited. A study from Alduaij et al. showed that GA101 (also named obinutuzumab), a novel type II anti-CD20 monoclonal antibody, can induce lysosomal cell death in lymphoma cell lines (Alduaij et al., 2011). Importantly, this type of GA101-induced lysosomal cell death can be abrogated by cytochalasin D and latrunculin B, inhibitors of actin polymerization. However, the detailed mechanism is unclear and further work is required to establish the missing link between lysosomal cell death and the actin cytoskeleton. Nevertheless, mounting evidences have indicated that lysosome activity correlates closely with the actin cytoskeleton. For example, early studies have shown that, depending on actin, overexpression of truncated myosin obstructs membrane transport from endosomes to preexisting lysosomes (van Deurs et al., 1995; Barois et al., 1998). Another report suggested that Myosin I Alpha (MMIα), a myosin associated with endosomes and lysosomes, acts in concert with actin filaments to mediate the delivery of internalized molecules to lysosomes and the movement of lysosomes can be disturbed by actin depolymerization agents (Cordonnier et al., 2001). One profound feature of lysosomal cell death is the release of Cathepsins. In other studies not directly related to lysosomal cell death, the actin machinery can be degraded by Cathepsins. Cathepsin B has a broad degradation effect on actin, with most of the peptides released from the N- and C- termini of the actin protein (Hughes et al., 1999). It has also been suggested that Cathepsin D can catalyze the hydrolysis of actin and myosin (Hughes et al., 2000). A recent study demonstrates that, during microglia migration, Cathepsin D can juxtapose actin filaments at the leading edge of lamellipodia through modulating the phosphorylation status of Cofilin (Liu et al., 2020). Moreover, Cathepsin L can degrade actin, actin regulatory proteins such as myosin heavy chain, Troponin T and Troponin I from rabbit skeletal muscle (Matsukura et al., 1981).

In conclusion, the actin machinery may play a role in lysosomal cell death but how exactly they impact the death signal transduction remains to be further investigated.

## Pyroptosis

**Figure 3:**
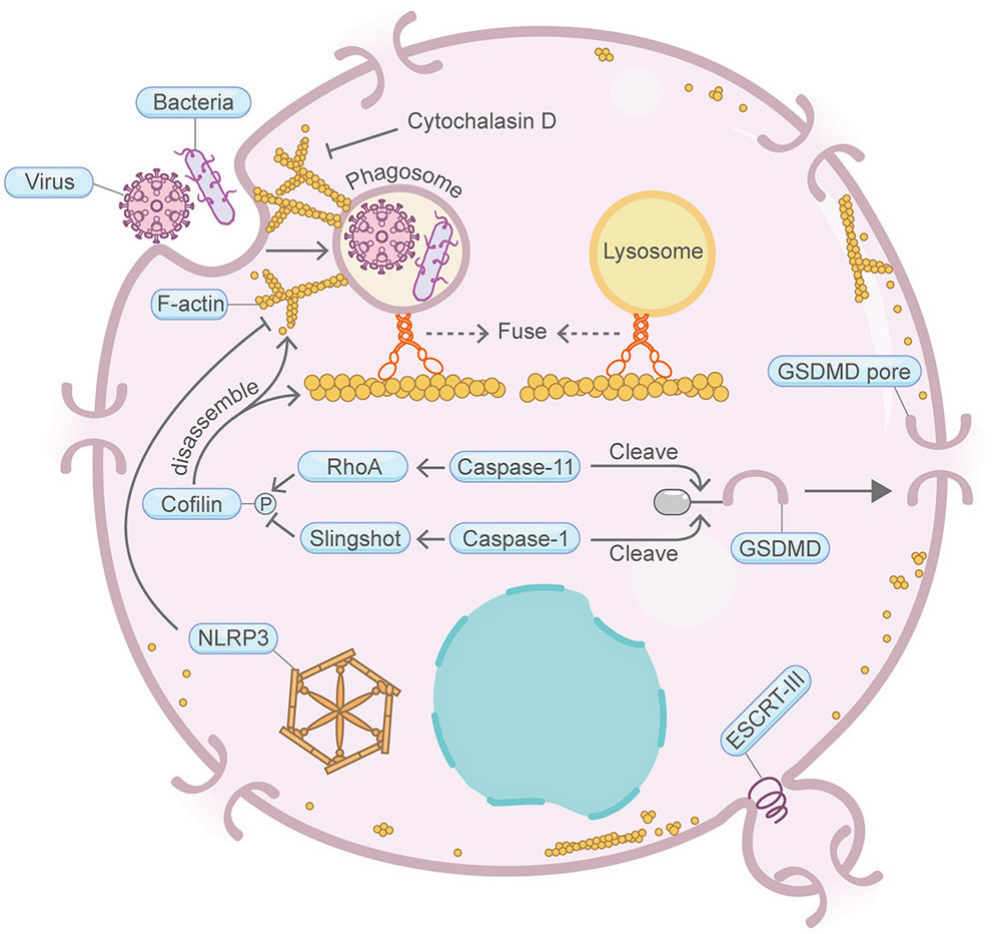
Schematic illustrating pyroptosis and the actin machinery. Actin machinery is required for pyroptosis. F-actin forms around phagosomes at the early stage of pyroptosis. Cortical actin networks are disrupted during the progression of pyroptosis. Caspase-11 and RhoA maintain the actin-severing protein Cofilin in the phosphorylated inactive form, which sustains actin polymerization and mediates phagosome-lysosome fusion. Cofilin activity is also regulated by Caspase-1 and Slingshot. NLRP3 inflammasome also represses F-actin remodeling. The ESCRT-III complex repairs damaged plasma membrane. ESCRT-III cooperates with the actin cytoskeleton during other processes such as cytokinesis and wound healing. Please see the main text for more detailed information. Abbreviations: GSDMD, gasdermin D; ESCRT, endosomal sorting complexes required for transport; NLRP3, NLR family pyrin domain containing 3; RhoA, Ras homolog family member A; Caspase, cysteine–aspartate protease.

Pyroptosis, a form of distinguished PCD initiated in response to infections, is characterized by the presence of phagosome formation, inflammasome assembly, GSDMD pore formation, cell swelling and release of inflammatory cytokines (Figure 3). Existing studies have shown that actin cytoskeleton is required for pyroptosis. At the early stage of pyroptosis, failure in forming the F-actin networks surrounding phagosomes in host cells causes prominent defects in pathogenic bacteria clearing (Fink and Cookson, 2006; Akhter et al., 2012). Treatment with the actin depolymerization agent cytochalasin D prevents rapid pyroptosis through inhibiting pathogen internalization (Fink and Cookson, 2007). Upon the progression of pyroptosis, the cortical F-actin network, together with microtubule and intermediate filaments, is gradually disrupted or cleaved (Davis et al., 2019). Meanwhile, Caspase-11, expressed broadly in immune and non-immune cells, modulates the fusion of phagosomes with lysosomes by regulating actin polymerization through the F-actin severing protein Cofilin (Abu Khweek and Amer, 2020). The phosphorylation status of Cofilin, which is modulated via the small GTP-binding protein RhoA or the phosphatase protein Slingshot (interact with Caspase-1), is directly linked to its F-actin severing activity during pyroptosis (Caution et al., 2015). This study also showed that Caspase-1 and Caspase-11, the two key regulators in pyroptosis, converge on the actin cytoskeleton in contrasting ways by dephosphorylating or phosphorylating Cofilin (Caution et al., 2015). Nevertheless, perturbation of actin dynamics with the actin depolymerization agent latrunculin A or the actin stabilization agent jasplakinolide does not impact the cell swelling feature of nigericin-induced pyroptosis (Davis et al., 2019). NLRP3 inflammasome is essential for pyroptosis. NLRP3 functions as an intrinsic inhibitory factor for virus entry by repressing F-actin remodeling (Paoletti et al., 2019). Intriguingly, it is noteworthy that the ESCRT-III complex, which cooperates with the actin cytoskeleton during cytokinetic abscission and wound healing (Meng et al., 2020; Vietri et al., 2020), functions in pyroptosis membrane repair (Rühl et al., 2018).

Together, these findings reveal a tight connection between pyroptosis and the actin machinery. However, it is still unclear whether more proteins in the actin machinery function in pyroptosis.

## NeTosis

**Figure 4:**
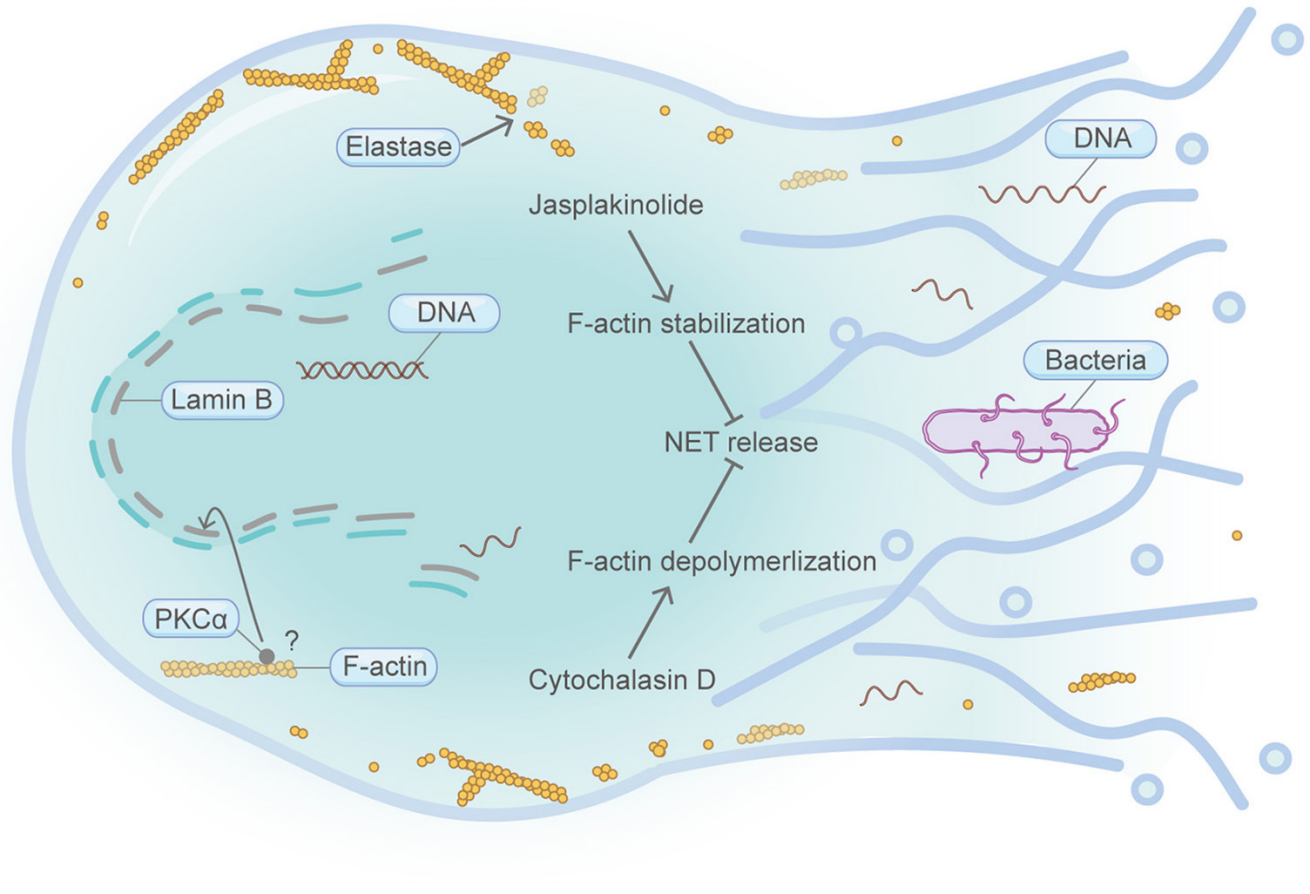
Schematic illustrating NETosis and the actin machinery. NETosis is exclusively found in neutrophils. Cortical F-actin disassembly is a prerequisite for NET release and occurs at the early stage of NETosis. Neutrophil elastase can bind and degrade F-actin network. Both F-actin stabilization by jasplakinolide and F-actin depolymerization by cytochalasin D lead to the attenuation of NET release. Execution of PKCα function in the nucleus is important for nuclear envelope rupture and NET formation. PKCα nuclear translocation requires intact actin cytoskeleton in NIH 3T3 fibroblasts. Please see the main text for more detailed information. Abbreviations: NET, neutrophil extracellular traps; PKCα, protein kinase C alpha.

NETosis, exclusively found in neutrophils, describes a highly specific cell death process involving a web-like DNA network (NET, neutrophil extracellular traps) decorated with chromatin fibers, histones and anti-microbial proteins (Figure 4). Cortical F-actin disassembly is a prerequisite for NET release and occurs at the early stage of NETosis (Gong et al., 2020; Thiam et al., 2020). Prior to the execution of NETosis, neutrophil elastase, before its translocation from the cytosol to the nucleus, binds and degrades actin network (Metzler et al., 2014). Additionally, it was reported that promoting the disassembly of the F-actin cytoskeleton by cytochalasin D dampens histone deimination and blocks NET release (Neeli et al., 2009). In these cells with impaired F-actin, neutrophil extracellular trap release is inefficient, despite the nuclear envelope has been broken down and the nucleoplasm contents are already mixed with its cytoplasmic counterparts. Similar results were also obtained with the F-actin stabilization agent jasplakinolide (Thiam et al., 2020). Stabilizing the F-actin cytoskeleton significantly reduced the percentage of cells that expelled extracellular traps. Moreover, PKCα-mediated phosphorylation of lamin B and subsequent nuclear envelope disassembly are important for the nuclear envelope rupture and NET formation during NETosis, wherein PKCα nuclear translocation in NIH 3T3 fibroblast requires intact actin cytoskeleton (Schmalz et al., 1996; Li et al., 2020). In addition, cyclin-dependent kinases CDK6, a key regulator of the cell cycle, is required for NETosis signaling (Albrengues et al., 2017). Coincidently, CDK6 associates with the actin cytoskeleton and is involved in the transcriptional regulation of a panel of actin regulatory genes (Uras et al., 2017).

Thus, emerging evidence supports the idea that the actin cytoskeleton is crucial for NETosis. However, it is currently unknown whether other components in the actin machinery engage actively in NETosis regulation. Further studies should be conducted to decipher the deeper relationship between NETosis and actin.

## Necroptosis

**Figure 5:**
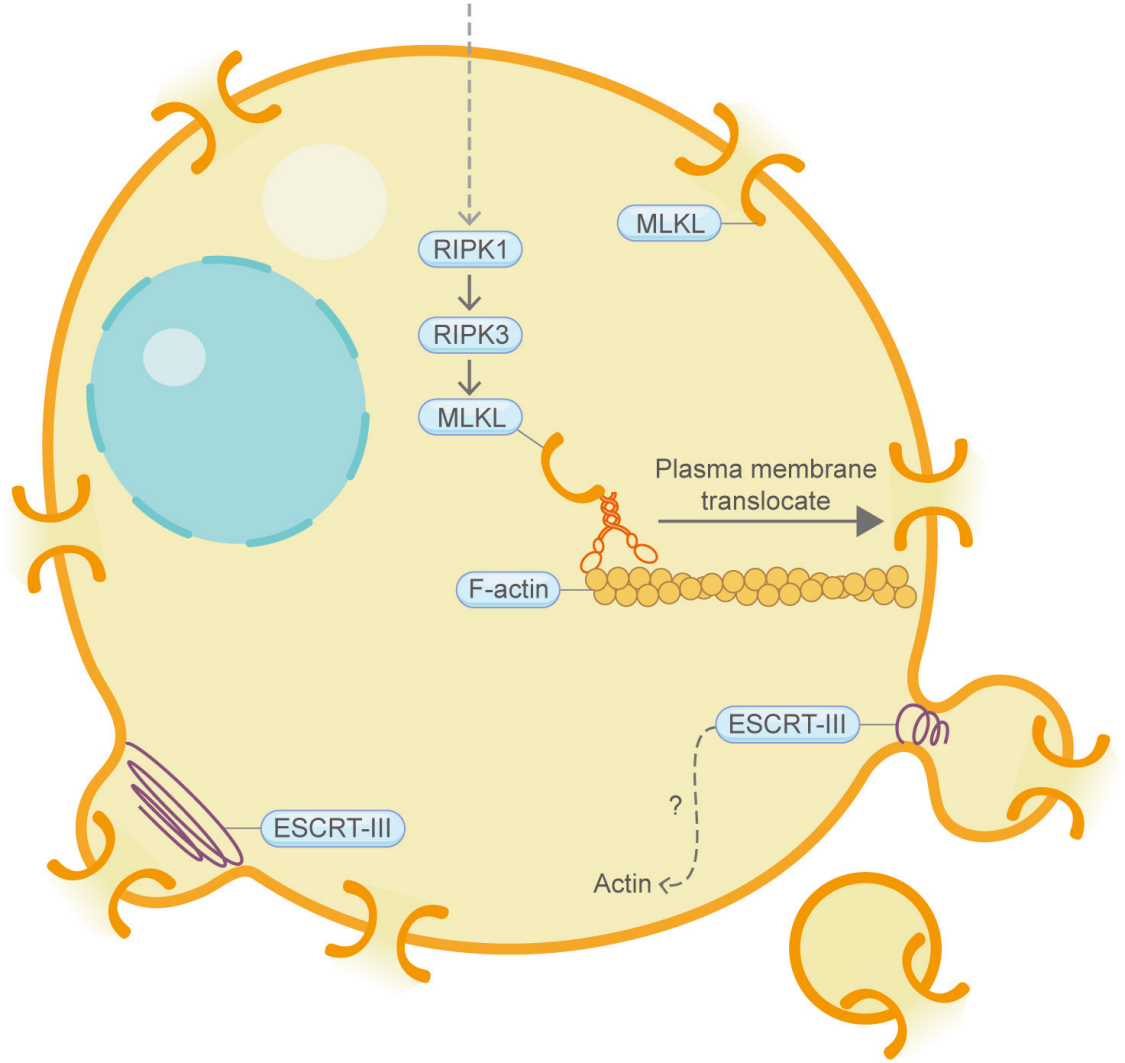
Schematic illustrating necroptosis and the actin machinery. MLKL is the terminal protein in necroptosis. During MLKL translocation to the plasma membrane, it co-traffics with tight junction proteins through Golgi-microtubule-actin-dependent mechanisms. The ESCRT-III complex, which coordinates with the actin cytoskeleton in other contexts, repairs damaged plasma membrane. Please see the main text for more detailed information. Abbreviations: MLKL, mixed lineage kinase domain-like pseudokinase; ESCRT, endosomal sorting complexes required for transport.

Necroptosis is a type of regulated necrosis that has similar morphological characteristics to canonical necrosis (Figure 5) (Vandenabeele et al., 2010). RIPK3, a key regulatory protein in necroptosis, can promote the oligomerization of the terminal protein MLKL and its translocation to the cell periphery, where MLKL ultimately leads to cell rupture. Although still under debate, the “point of no return” of necroptosis has often been attributed to MLKL activation (Gong et al., 2019). Strikingly, a recent study utilizing the actin depolymerization agent cytochalasin B showed that, during MLKL translocation to the plasma membrane, it co-traffics with tight junction proteins through Golgi-microtubule-actin-dependent mechanisms (Samson et al., 2020). During macrophage necroptosis, cell-to-cell transfer of the fungi, *Aspergillus fumigatus*, is based on F-actin-dependent exocytosis (Shah et al., 2016). In addition, continued downregulation of Villin-1 and Gelsolin, two actin-binding proteins, can downregulate cytoplasmic G-actin level and inhibit PP1 phosphatase activity, thereby leading to constitutive phosphorylation of EIF2A and subsequently upregulation of IRGM1, which induces necroptosis probably through affecting mitochondria and autophagy (Roy et al., 2018). Similar to what is mentioned in pyroptosis, the ESCRT-III complex functions downstream of MLKL, thereby facilitating the shedding of MLKL-induced damaged plasma membrane and antagonize necroptotic cell death (Gong et al., 2017). F-actin depolymerizes extensively and quickly in tumor cells upon immunological synapse formation between natural killer cells and MCF7 cells that are destined for necroptosis. Interestingly, during the process of cell clearance by macrophages, necroptosis cells present high levels of CD47 on the cell surface, which can induce RhoA-pMLC signaling in macrophages that hinders the whole-cell engulfment of necroptosis cells (Gerlach et al., 2020).

Together, these findings have revealed a growing connection between necroptosis and the actin machinery.

## Entosis

**Figure 6:**
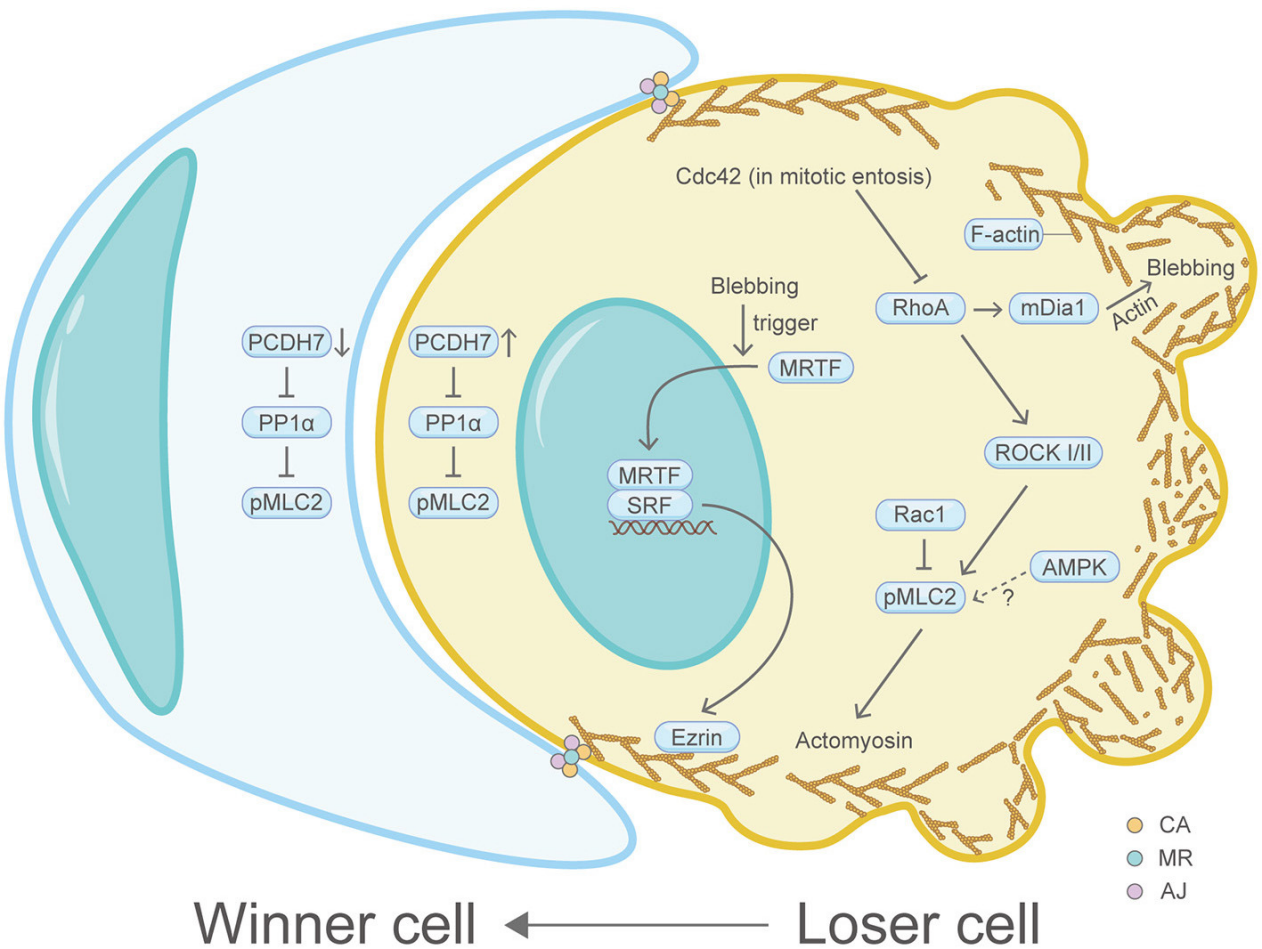
Schematic illustrating entosis and the actin machinery. Actin machinery is essential for entosis. In the loser cell, signaling pathway proteins including Cdc42, RhoA, ROCK I/II, Rac1, mDia1, AMPK, PCDH7, PP1α, MRTF, SRF, and Ezrin converge on the actomyosin network to regulate entosis. In the winner cell, PCDH7 and PP1α also exist. Three actin-correlated structures, the contractile actomyosin, the adherens junction and the mechanical ring, are sandwiched between the winner cell and the loser cell. Please see the main text for more detailed information. Abbreviations: Cdc42, cell division control protein 42; RhoA, Ras homolog family member A; ROCK, Rho-associated coiled-coil kinase; Rac1, Ras-related C3 botulinum toxin substrate 1; pMLC2, phosphorylated myosin light chain 2; AMPK, AMP-activated protein kinase; mDia1, diaphanous-related formin 1; PCDH7, protocadherin-7; PP1α, protein phosphatase 1α; MRTF, myocardin-related transcription factor; SRF, serum response factor; CA, the contractile actomyosin; MR, the mechanical ring; AJ, the adherens junction.

Entotic cell death (entosis), a programmed cell cannibalism, dictates a cell engulfment process that a loser cell (invading cell/internalizing cell) invades and is generally killed by its neighboring cell (winner cell/host cell/engulfing cell) through a mechanism involving autophagy proteins and lysosomal enzymes, however, without caspase activation (Figure 6) (Overholtzer et al., 2007). Matrix detachment, glucose starvation and mitosis can induce entosis, which may kill matrix-detached tumor cells or promote cancer cell polyploidy formation (Krajcovic et al., 2011). A flurry of studies have demonstrated that the actin machinery is utmost important for the winner or loser identity of cells (Sun et al., 2014b). For example, sustained plasma membrane blebbing is vital for the entotic invasion process in the loser cells, which functions through the MRTF-SRF-Ezrin axis (Grosse et al., 2017). In line with this, plasma membrane blebbing is found to be inseparable with F-actin and myosin functions (Chikina et al., 2019). A recent study showed the invading cell produces projections at its rear end which contain the actin-binding protein mDia1 (Purvanov et al., 2014). Moreover, IL-8 was identified as a positive regulator of homotypic entotic cell-in-cell (CIC) formation (Ruan et al., 2018). Coincidentally, IL-8 can promote F-actin polymerization in U87 cells (Zhang et al., 2015). Consistently, the AMP-activated protein kinase (AMPK), which is important for stiffness regulation and autophagy regulation in loser cells, can also induce actin cytoskeleton reorganization (Hamann et al., 2017; Schubert et al., 2017). Another study showed that the cell division control protein Cdc42 can regulate RhoA, thereby regulating the actin cytoskeleton-dependent mitotic entosis (Durgan et al., 2017). Similarly, Rac1 can regulate myosin light chain 2 (MLC2) phosphorylation to modulate entosis (Sun et al., 2014b). Consequently, actomyosin, the downstream effector of RhoA signaling, is highly enriched and activated at the rear cortex of the invading cell, therefore driving cell internalization (Wang et al., 2020b).

Recently, it has been recognized that three core compartmentalized ring-like structures are assembled and sandwiched between the winner and loser cells: the contractile actomyosin, the adherens junction and the mechanical ring (Wang et al., 2020b). Multiple actin machinery proteins such as E- or P-cadherin, multiple essential catenins, junction localized-p190A RhoGAP, RhoA, ROCK I/II, pMLC2, MHC IIA and IIB and actin, constitute these ring-like complexes and are proved to accumulate at high levels (Sun et al., 2014a). At the cell-cell contact site, the transmembrane protein PCDH7 can positively regulate the actin machinery protein pMLC2 by inactivating protein phosphatase 1α (PP1α), thereby increasing actomyosin contraction (Wang et al., 2020a). Additionally, the contractile actomyosin ring was further connected with a dome-like structure formed by cortex F-actin and MLC at the rear region of the invading cell (Wang et al., 2020b). The mechanical sensor vinculin in the mechanical ring detects mechanical forces imposed on cells and serves as a compartmentalizing factor to promote entosis. Perturbation of vinculin compromises entosis (Wang et al., 2020b). Interestingly, it was shown in other physiological contexts that vinculin can change F-actin localization or dynamics by recruiting F-actin filaments to the growing focal adhesions or capping actin filament barbed ends (Golji and Mofrad, 2013).

In general, these data underline the fundamental roles of the actin machinery in entosis.

## Parthanatos

**Figure 7:**
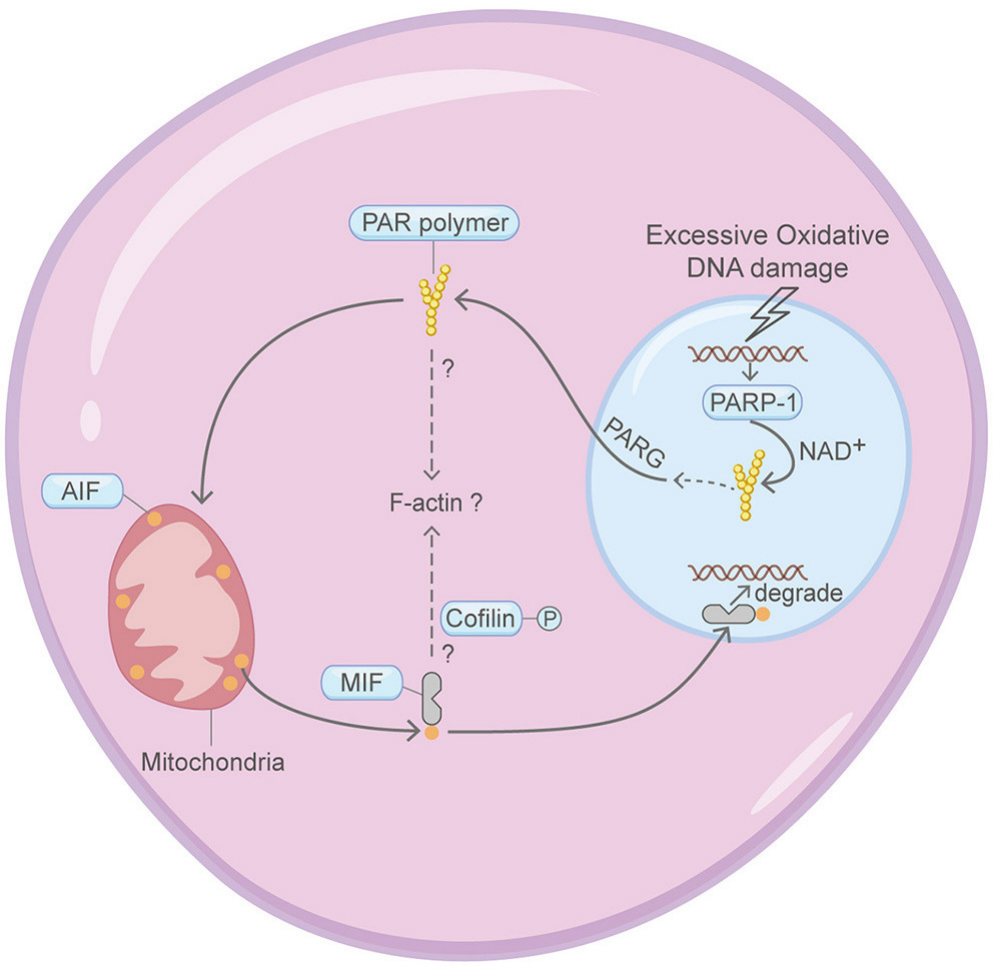
Schematic illustrating parthanatos and the actin machinery. It is unclear whether the actin machinery is directly involved in parthanatos. MIF and PARP family proteins are found to affect the actin cytoskeleton in other contexts. Please see the main text for more detailed information. Abbreviations: PAR, poly(ADP-ribose); PARP-1, poly(ADP-ribose) polymerase 1; MIF, migration inhibitory factor; AIF, apoptosis-inducing factor; NAD^+^, nicotinamide adenine dinucleotide; PARG, poly(ADP-ribose) glycohydrolase.

Parthanatos, which depends on PARP-1 activation, PAR signaling and mitochondrial AIF translocation, refers to a form of cell death pivotal in multiple neural diseases (Figure 7) (Berger et al., 1983; Andrabi et al., 2008; David et al., 2009; Kam et al., 2018). Four crucial steps are involved in the commitment of parthanatos: PARP-1 activation, PAR polymer assembly, mitochondrial apoptosis-inducing factor AIF release, AIF-mediated chromatin condensation and DNA fragmentation (Wang et al., 2009; Robinson et al., 2019). The connection between parthanatos and the actin machinery is poorly explored. But interestingly, cytoplasmic PARP family proteins are found to regulate the actin cytoskeleton in other studies (De Lisa et al., 2012; Vyas et al., 2013). Moreover, the migration inhibitory factor (MIF), a cytoplasmic endonuclease that co-translocates with AIF to the nucleoplasm and induces DNA fragmentation, may affect F-actin dynamics through regulating the phosphorylation status of Cofilin (Hu et al., 2015; Wang et al., 2016).

Thus, there is limited knowledge about whether the actin machinery is directly involved in the regulation of parthanatos.

## Ferroptosis

**Figure 8:**
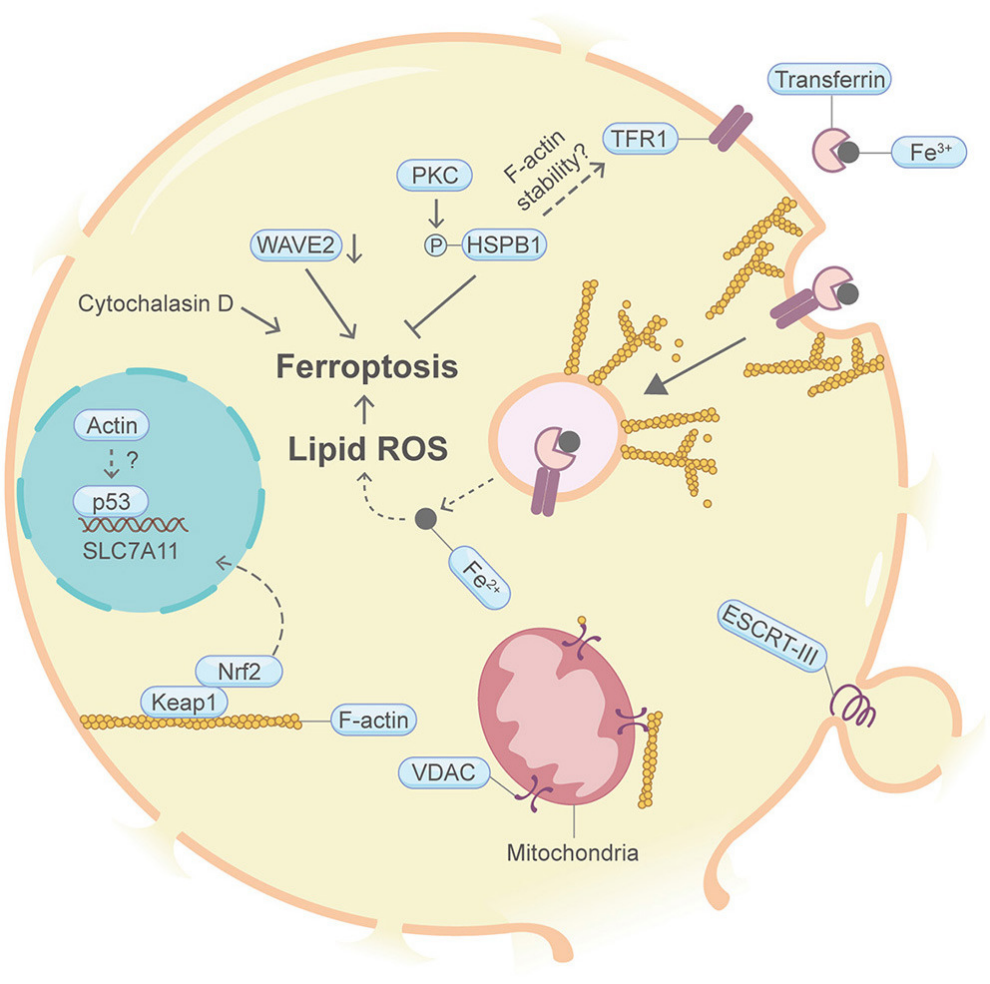
Schematic illustrating ferroptosis and the actin machinery. PKC-HSPB1 attenuates ferroptosis. HSPB1 regulates F-actin cytoskeleton and TFR1 in other contexts. Cytochalasin D, which depolymerize F-actin, promotes ferroptosis. Suppression of WAVE2, which regulates the branched F-actin network assembly in other studies, favors ferroptosis. Nrf2, p53, and VDAC may be modulated by actin. The ESCRT-III complex, which coordinates with the actin cytoskeleton in other contexts, repairs damaged plasma membrane. Please see the main text for more detailed information. Abbreviations: HSPB1, heat shock protein beta-1; PKC, protein kinase C; WAVE2, Wiskott-Aldrich Syndrome protein family member 2; Keap1, Kelch-like ECH-associated protein 1; VDAC, voltage-dependent anion channel; ESCRT, endosomal sorting complexes required for transport.

Ferroptosis is a form of programmed necrotic cell death with high lipid peroxidation as its leading feature (Figure 8). The death mechanism of ferroptosis is still obscure. It is proposed that high lipid peroxidation causes postulated membrane distortion, thereby leads to cell permeabilization, possibly without pore-forming effector proteins. It is largely unclear whether the actin machinery is actively engaged in ferroptosis. A handful of studies may hint this possible connection. For example, knocking down the heat shock protein beta-1 (HSPB1, also named HSP27) or suppression of HSPB1 phosphorylation by protein kinase C (PKC) inhibitors can enhance erastin-induced ferroptosis (Sun et al., 2015). In other contexts, HSPB1 can downregulate TFR1-mediated iron uptake by stabilizing the F-actin cytoskeleton (Lavoie et al., 1993; Rousseau et al., 1997; Chen et al., 2006). Moreover, disruption of the actin cytoskeleton by the F-actin depolymerization agent cytochalasin D increases intracellular iron level, membrane lipid peroxidation and decreases cell viability (Sun et al., 2015). Furthermore, suppression of WAVE2, the upstream key member of Wiskott–Aldrich syndrome protein regulating the branched F-actin network assembly, increased intracellular iron and exhibited growth retardation following erastin treatment (Sun et al., 2015). The transcription factor Nrf2 plays key roles in antagonizing ferroptosis (Fan et al., 2017). In other studies not directly connected with ferroptosis, the nuclear translocation of Nrf2 was shown to be regulated by the F-actin-Keap1 axis (van Der Kammen et al., 2017). Additionally, the mitogen-activated protein kinase (MAPK) pathway contributes to ferroptosis (Poursaitidis et al., 2017). Coincidently, activation of the MAPK pathway is closely linked to the actin cytoskeleton (Tsakiridis et al., 1998; Tomas et al., 2006). p53, the guardian of the genome, is well-known to interact with actin. Interestingly, p53 plays dual roles in ferroptosis through inhibiting the transcription of the key system Xc^−^ gene SLC7A11 (Kang et al., 2019). Through FOXM1 and Nedd4, protein levels of VDAC2 and VDAC3 decrease after erastin-induced ferroptosis (Yang et al., 2020). As mentioned in apoptosis, VDAC is known to be regulated by cytoplasmic actin dynamics (Gourlay and Ayscough, 2005). Last but not the least, as with pyroptosis and necroptosis, the ESCRT-III complex repairs membrane in ferroptosis (Dai et al., 2020).

Nevertheless, it remains unknown whether the actin cytoskeleton and its regulatory proteins are highly involved in the ferroptosis process.

## Autosis

**Figure 9:**
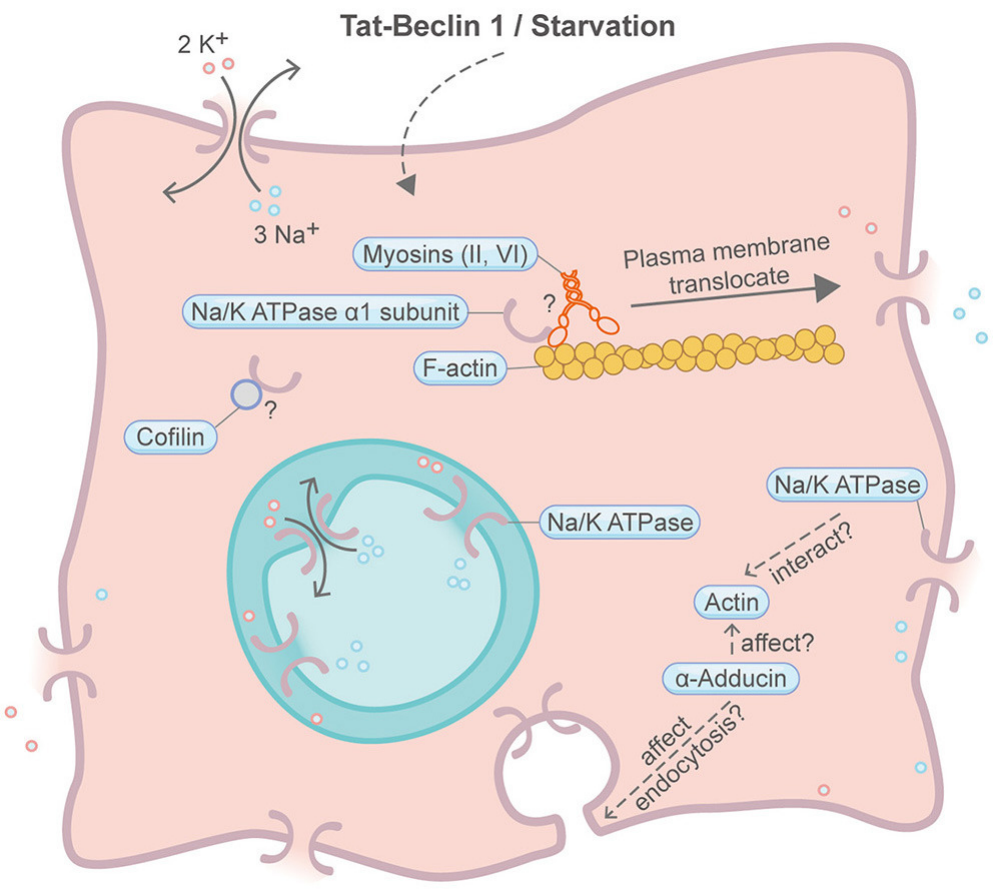
Schematic illustrating autosis and the actin machinery. It is unclear whether the actin machinery is directly involved in autosis. In other contexts, Na^+^,K^+^-ATPase is found to interact with actin, Cofilin and multiple myosin motors. Na^+^,K^+^-ATPase may also be affected by the actin cytoskeleton through α-adducin. Please see the main text for more detailed information. Symbols: K^+^, potassium ion; Na^+^, sodium ion.

Autosis is a specific form of autophagy-dependent cell death induced by starvation or an autophagy-inducing cell-permeable peptide, Tat-Beclin 1 (Figure 9) (Liu et al., 2013; Zhang et al., 2015; Fernandez et al., 2020). Autosis is also found in rat hippocampal neurons subjected to hypoxic–ischemic injury, patients with anorexia nervosa and animal models of renal ischemia (Liu et al., 2013; Fernandez et al., 2020). Autophagy plays fundamental roles in this type of PCD. A comprehensive review about autophagy and the actin cytoskeleton has been described elsewhere (Kast and Dominguez, 2017). A milestone for autosis is the discovery of the integral membrane Na^+^,K^+^-ATPase pump by chemical screening of autosis inhibitors (Liu et al., 2013). Suppression of Na^+^,K^+^-ATPase by cardiac glycosides efficiently rescue autotic cell death. Intriguingly, it was recently found that the physical interaction between Na^+^,K^+^-ATPase and the autophagy protein Beclin 1 is essential for autosis (Fernandez et al., 2020). To the best of our knowledge, there is no evidence showing direct involvement of the actin machinery in autosis. However, an earlier study suggested that actin can bind and stimulate the Na^+^,K^+^-ATPase pump (Cantiello, 1995). In addition, Na^+^,K^+^-ATPase alpha 1 subunit may interact with the F-actin severing protein Cofilin (Lee et al., 2001). Moreover, actin may indirectly impact Na^+^,K^+^-ATPase plasma membrane retention through a protein called α-Adducin, which affects endocytosis and actin polymerization (Torielli et al., 2008). Recently, multiple myosin motors (myh9, myh10, myh14, and myoVI) are also found to interact with the Na^+^,K^+^-ATPase alpha 1 subunit (Dash et al., 2018). Interestingly, Na^+^,K^+^-ATPase presents both on the plasma membrane and on the inner nuclear membrane (Garner, 2002; Galva et al., 2012).

In all, the participation of the actin machinery in autosis remains inconclusive, but it is worth more efforts to dig deeper into their relationship.

## Alkaliptosis

Most recently, a promising PCD called “Alkaliptosis” was discovered, which is caused by intracellular alkalinisation (Song et al., 2018). Due to its very recent discovery, it is yet unclear whether the actin machinery is enrolled in alkaliptosis, hence is not covered in this review. In view of the connections between other PCDs and actin, it is reasonable to speculate if the actin machinery also plays a role in alkaliptosis.

## Oxeiptosis

**Figure 10:**
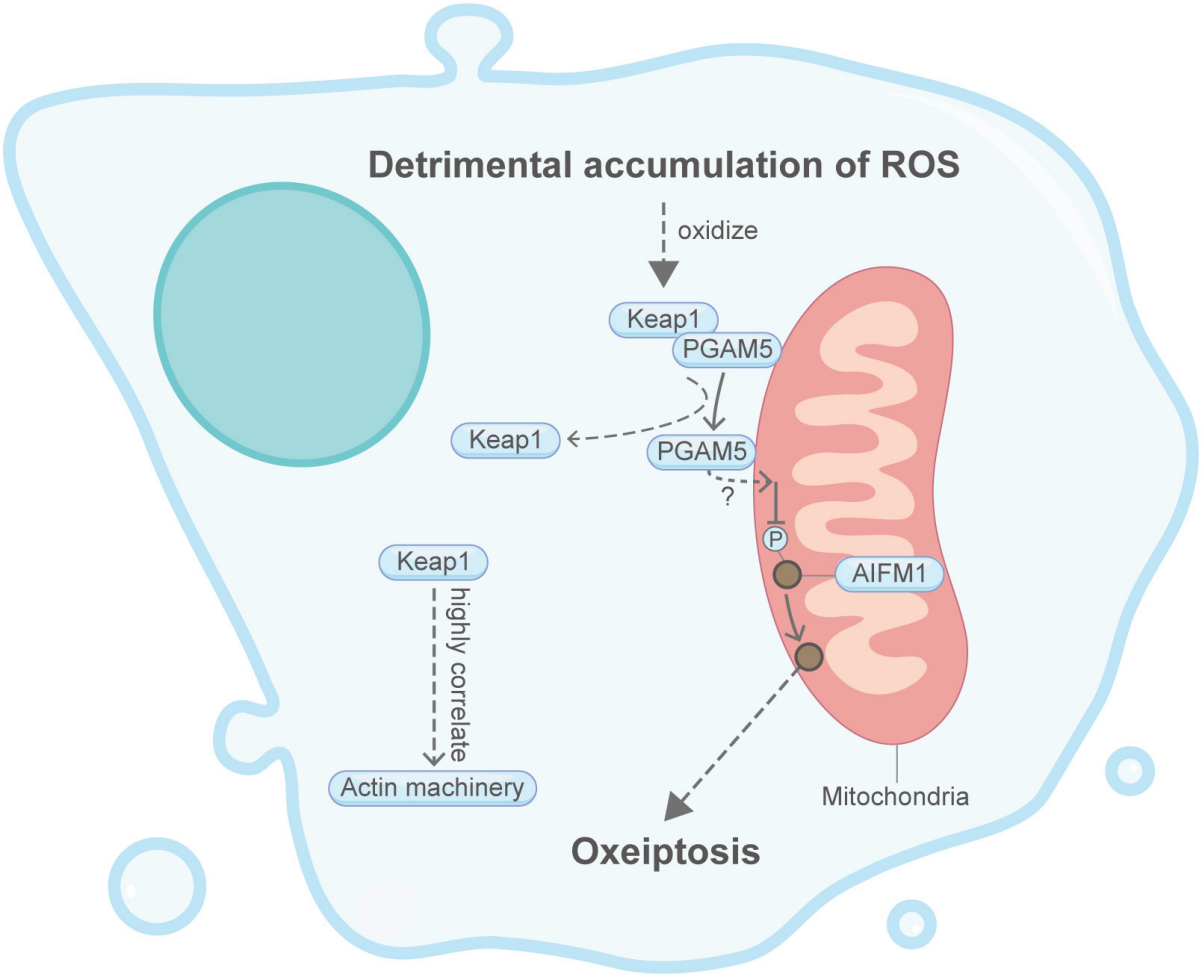
Schematic illustrating oxeiptosis and the actin machinery. It is unclear whether the actin machinery is directly involved in oxeiptosis. Keap1, an essential protein in oxeiptosis, highly correlates with the actin machinery in other contexts. Please see the main text for more detailed information. Abbreviations: Keap1, Kelch-like ECH-associated protein 1; PGAM5, PGAM family member 5; AIFM1, apoptosis-inducing factor mitochondria associated 1.

Oxeiptosis, initially found from an *in vivo* ozone-exposure mice model, is considered an anti-inflammatory form of regulated cell death in response to toxic levels of ROS (Figure 10) (Holze et al., 2018; Scaturro and Pichlmair, 2018). During oxeiptosis, detrimental accumulation of ROS was shown to oxidize Keap1, which then uncouples with the mitochondrial membrane protein PGAM5 (Lo and Hannink, 2008; Scaturro and Pichlmair, 2018). PGAM5 in turn binds and dephosphorylates AIFM1 (also known as AIF1) and eventually leads to the progression of a caspase-independent oxeiptosis cell death. Although not directly related, it is found in other studies that, under resting conditions, the intracellular ROS sensor Keap1 interacts and colocalizes with the cytosolic F-actin cytoskeleton via its DGR/Kelch region (Kopacz et al., 2020). Moreover, overexpression of Keap1 can stabilize and reorganize cytosolic F-actin through evident Myo9b downregulation (Wu et al., 2018). Additionally, Keap1 can affect actin machinery proteins such as myosin VIIa, cortactin and RhoGAP1 (Kopacz et al., 2020). Intriguingly, H_2_O_2_-induced oxeiptosis cells also showed membrane blebbing (Holze et al., 2018), whose relationship with the actin machinery has been described in apoptosis and entosis above.

Together, these limited studies indicate that oxeiptosis could be well-associated with the actin machinery. However, due to the short discovery time from 2018, the direct relationship between oxeiptosis and the actin cytoskeleton remains underexplored.

## Conclusion

Programmed cell death plays instrumental and indispensable roles in embryonic development and disease progression in living organisms. To date, more than eleven kinds of PCDs are identified. However, the detailed mechanisms of most PCDs remain largely unclear. Different forms of PCD possess distinct signaling pathways, but interestingly, some of them may converge at a few common regulators. The actin cytoskeleton, a highly conserved and dynamic core cellular machinery, plays pivotal roles in a plethora of cellular processes, including morphogenesis, differentiation, cell motility, cell division, cytokinesis, membrane trafficking, etc. In the present review, we focus on the links between PCDs and the actin machinery proteins (Table 1). Clear and strong facts linking the actin machinery and apoptosis, pyroptosis, NETosis, necroptosis, and entosis are summarized. A handful of evidence indicates that the actin machinery could be connected with lysosomal cell death and ferroptosis. The direct relationships between the actin machinery and parthanatos, autosis, oxeiptosis, and alkaliptosis require further investigation. Under different contexts of PCD, the actin machinery may affect mitochondria, intracellular vesicle transport, vesicle fusion, cytoplasmic protein retention, receptor internalization, membrane deformability, ion channel, membrane repair, endocytosis, protein phosphorylation, etc. It should be emphasized that the actin cytoskeleton may play opposing roles in different PCDs, either by promoting or inhibiting cell death (e.g., apoptosis and necroptosis). Furthermore, it would be beneficial to distinguish whether the actin machinery really plays critical decision-making roles in each PCD or it is merely an outcome of the PCD process. In general, the relationship between PCDs and actin cytoskeleton is still in its infancy.

**Table 1:** Actin machinery and PCDs.

	**Score**	**Actin machinery**	**Direct force production**	**Membrane repair**	**Transport**	**Exocytosis endocytosis**	**Blebbing**	**Chemicals**
Apoptosis	√√√	Actin, Myosin, Caspase, α-Actinin, Gelsolin, β-Thymosins, Filamin, E-tropomodulin, Coronin-1, RhoA, Rac-1, Cofilin, WAVE1, Villin, DNase I, ROCK1, Par-4, Dlk, DR4/DR5, TNF receptor-1, CD95/Fas, CD44, Ezrin, VDAC, p53, E-cadherin, Catenins, Dlc2	Actin, Myosin	N.A.	Actin, Myosin	N.A.	Actin, myosin, ROCK1, Gelsolin, Caspase	Cytochalasin D, Latrunculin A, Jasplakinolide, Simvastatin, Phalloidin
Lysosomal cell death	√	Actin, Myosin, Cathepsin B, Cathepsin D, Cathepsin L, Cofilin, Troponin	N.A.	N.A.	N.A.	N.A.	N.A.	GA101, Cytochalasin D, Latrunculin B
Pyroptosis	√√√	Actin, Myosin, Cofilin, RhoA, Slingshot, ESCRT-III complex, Caspase, NLRP3	N.A.	ESCRT-III	N.A.	N.A.	N.A.	Cytochalasin D
NETosis	√√√	Actin, neutrophil elastase, PKCα, CDK6	N.A.	N.A.	N.A.	N.A.	N.A.	Cytochalasin D, Jasplakinolide
Necroptosis	√√√	Actin, Myosin, Villin-1, Gelsolin, ESCRT-III complex	N.A.	ESCRT-III	Actin	Actin	N.A.	Cytochalasin B
Entosis	√√√	Actin, Myosin, MRTF, SRF, Ezrin, Cdc42, RhoA, mDia1, ROCK I/II, Rac1, AMPK, Cadherin, Catenins, p190A RhoGAP, Vinculin, PCDH7, PP1α	Actin, Myosin, Cdc42, RhoA, Rac1, ROCK, Vinculin	N.A.	N.A.	N.A.	Actin, Myosin, MRTF, SRF, Ezrin, Cdc42, RhoA, mDia1, ROCK I/II, Rac1, AMPK	N.A.
Parthanatos	^*^	Actin, MIF, Cofilin, PAR	N.A.	N.A.	N.A.	N.A.	N.A.	N.A.
Ferroptosis	√	Actin, HSPB1, PKC, WAVE2, Nrf2, Keap1, MAPK, p53, VDAC, ESCRT-III complex	N.A.	ESCRT-III	N.A.	Actin	N.A.	Cytochalasin D
Autosis	^*^	Na^+^,K^+^-ATPase, Cofilin, α-Adducin, Myosin	N.A.	N.A.	N.A.	Na^+^,K^+^-ATPase, α-Adducin	N.A.	N.A.
Alkaliptosis	^*^	N.A.	N.A.	N.A.	N.A.	N.A.	N.A.	N.A.
Oxeiptosis	^*^	Actin, Keap1, Myosin	N.A.	N.A.	N.A.	N.A.	Actin, myosin	N.A.

√√√ Strong connection;

√ Weak connection;

* *To be investigated. Some actin machinery proteins may not directly participate in PCDs. Please refer to the main text for detailed information*.

It is noteworthy that actin and a number of proteins involved in the actin machinery exist both in the cytoplasm and the nucleoplasm. While the cytoplasmic actin machinery claims its emerging role in PCDs, it remains mostly unclear whether the nuclear actin machinery is involved. Therefore, research surrounding the connections between PCDs and the nuclear actin machinery should be a high priority for exploration in the future.

Most of the current observations have so far employed techniques dissecting the actin machinery and PCDs in fixed cells. However, life is not static. Future research should utilize time-lapse imaging to unravel the dynamic spatial-temporal relationship between the actin machinery and different PCDs in live cells.

Readers should also notice that the discoveries about PCDs and the actin machinery mentioned in this review may be specific in some cell types or under specific conditions. Even the same actin depolymerization agent treatment may result in contrasting cell fates in different types of cells (Paul et al., 2002; Kim et al., 2003). Meanwhile, some actin machinery proteins may be indirectly linked to PCDs, hence their actual roles in PCDs remain to be explored. In view of this, further studies should be conducted to investigate whether the actin machinery plays a universal role in these PCDs and how exactly they function in the PCD apparatuses. Currently, it is largely unclear how different PCDs interplay and communicate under complex cell death-inducing conditions. Deciphering the mechanism of the actin machinery may help answer this question.

Taken together, based on the studies mentioned above and given the profound roles the actin machinery plays in different PCDs, we hope that this review will stimulate further studies, and that bold guesses and hypotheses can be inspired and more secrets about PCDs and the actin machinery will be explored.

## Author Contributions

WR, WZ, LC, and JH: conceptualization and writing—original draft preparation. JH: funding acquisition. All authors contributed to the article and approved the submitted version.

## Conflict of Interest

The authors declare that the research was conducted in the absence of any commercial or financial relationships that could be construed as a potential conflict of interest.
